# Deficiency of TDAG51 Protects Against Atherosclerosis by Modulating Apoptosis, Cholesterol Efflux, and Peroxiredoxin‐1 Expression

**DOI:** 10.1161/JAHA.113.000134

**Published:** 2013-06-21

**Authors:** Gazi S. Hossain, Edward G. Lynn, Kenneth N. Maclean, Ji Zhou, Jeffrey G. Dickhout, Šárka Lhoták, Bernardo Trigatti, John Capone, Jaerang Rho, Damu Tang, Christopher A. McCulloch, Imtisal Al‐Bondokji, Mary J. Malloy, Clive R. Pullinger, John P. Kane, Yonghong Li, Dov Shiffman, Richard C. Austin

**Affiliations:** 1 Division of Nephrology, Department of Medicine, McMaster University and St. Joseph's Healthcare Hamilton, Ontario, Canada (G.S.H., E.G.L., J.Z., J.G.D., L., D.T., I.A.B., R.C.A.); 2 Department of Pediatrics, School of Medicine, University of Colorado Health Sciences Center, Aurora, CO (K.N.M.); 3 Department of Biochemistry and Biomedical Sciences, McMaster University, Hamilton, Ontario, Canada (B.T.); 4 Office of the Vice‐President (Research), Western University, London, Ontario, Canada (J.C.); 5 Department of Microbiology and Molecular Biology and GRAST, Gung‐dong, Yuseong‐gu, Daejeon, Korea (J.R.); 6 Matrix Dynamics Group, University of Toronto, Toronto, Ontario, Canada (C.A.M.C.); 7 Cardiovascular Research Institute, University of California, San Francisco, CA (M.J.M., C.R.P., J.P.K.); 8 Celera, 1401 Harbor Bay Parkway, Alameda, CA (Y.L., D.S.)

**Keywords:** apoptosis, arteriosclerosis, atherosclerosis, cardiovascular diseases

## Abstract

**Background:**

Apoptosis caused by endoplasmic reticulum (ER) stress contributes to atherothrombosis, the underlying cause of cardiovascular disease (CVD). T‐cell death‐associated gene 51 (TDAG51), a member of the pleckstrin homology‐like domain gene family, is induced by ER stress, causes apoptosis when overexpressed, and is present in lesion‐resident macrophages and endothelial cells.

**Methods and Results:**

To study the role of *TDAG51* in atherosclerosis, male mice deficient in TDAG51 and *apolipoprotein E* (*TDAG51*^*−/−*^/*ApoE*^*−/−*^) were generated and showed reduced atherosclerotic lesion growth (56±5% reduction at 40 weeks, relative to *ApoE*^*−/−*^ controls, *P*<0.005) and necrosis (41±4% versus 63±8% lesion area in *TDAG51*^*−/−*^*/ApoE*^*−/−*^ and *ApoE*^*−/−*^, respectively; *P*<0.05) without changes in plasma levels of lipids, glucose, and inflammatory cytokines. TDAG51 deficiency caused several phenotypic changes in macrophages and endothelial cells that increase cytoprotection against oxidative and ER stress, enhance PPARγ‐dependent reverse cholesterol transport, and upregulate peroxiredoxin‐1 (Prdx‐1), an antioxidant enzyme with antiatherogenic properties (1.8±0.1‐fold increase in Prdx‐1 protein expression, relative to control macrophages; *P*<0.005). Two independent case–control studies found that a genetic variant in the human TDAG51 gene region (rs2367446) is associated with CVD (OR, 1.15; 95% CI, 1.07 to 1.24; *P*=0.0003).

**Conclusions:**

These findings provide evidence that *TDAG51* affects specific cellular pathways known to reduce atherogenesis, suggesting that modulation of *TDAG51* expression or its activity may have therapeutic benefit for the treatment of CVD.

## Introduction

Cardiovascular disease (CVD) is an acute clinical manifestation of atherothrombosis that accounts for the majority of deaths in North America.^1^ A number of risk factors are known to accelerate CVD, including hypercholesterolemia, smoking, diabetes, hypertension, hyperhomocysteinemia, and obesity. Despite the diversity of these risk factors, the development and progression of atherosclerotic lesions is remarkably similar. Endothelial cell dysfunction and the accumulation of cholesterol‐rich lipoproteins in the vessel wall are early events in atherogenesis, resulting in the recruitment of circulating monocytes, their adhesion to the endothelium, their subsequent differentiation into macrophages, and the accumulation of lipid to form foam cells.^2^ In humans, these fatty streaks can progress to more advanced lesions characterized by a lipid‐rich necrotic core and a fibrous cap consisting of smooth muscle cells and collagen.

The acute clinical manifestations of atherosclerosis result from plaque rupture, thrombus formation, and vessel occlusion.^3^ Apoptotic cell death is a key feature of unstable plaques^4^ and is induced by a number of cellular stress pathways, including oxidative and endoplasmic reticulum (ER) stress.^5–7^ The distribution of cell death is heterogeneous within advanced lesions, but is most prominent in the lipid‐rich necrotic core that contains a high density of macrophages. Apoptotic cell death increases the risk of plaque rupture by decreasing the number of viable smooth muscle cells necessary for collagen production and compromising the structural integrity of the fibrous cap following release of matrix metalloproteinases from dead macrophages.^8^ Furthermore, plaque thrombogenicity is enhanced because lesion‐resident cells undergoing apoptosis express active cell surface tissue factor (TF),^9^ the major physiological initiator of the coagulation cascade.^10^ Previous studies have demonstrated that the absence of specific proapoptotic factors such as Bax^11^ or Rb^12^ decreases macrophage apoptosis as well as necrotic core size in hyperlipidemic mice. Consistent with these findings, a reduction in apoptosis and plaque necrosis was observed in advanced atherosclerotic lesions from *ApoE*^*−/−*^ mice deficient in the ER stress effector CHOP.^5^

*TDAG51* is a member of the pleckstrin homology‐like domain family having proapoptotic characteristics.^13^ Furthermore, *TDAG51* is induced by ER stress,^14–16^ and its overexpression in human vascular endothelial cells induces apoptotic cell death by disrupting cytoskeletal structure and impairing cell adhesion.^15^ Conversely, deficiency of TDAG51 contributes to apoptosis resistance and growth dysregulation in metastatic melanomas in vivo.^17^ *TDAG51* can also regulate energy metabolism by modulating adipogenesis and hepatic lipogenesis, which correlates with mature‐onset metabolic disease.^18^ Several lines of evidence implicate *TDAG51* in atherosclerotic lesion development. TDAG51 expression is increased in lesion‐resident macrophages and endothelial cells during all stages of atherogenesis.^6,15^ Furthermore, *TDAG51* mRNA is significantly increased in cultured human vascular endothelial cells following exposure to athero‐prone waveform stimulation.^19^ Although these findings suggest that TDAG51 contributes to the atherosclerotic process, it is currently unknown if TDAG51 is causally related to atherogenesis or if its mechanism of action stems from its previously described role as a proapoptotic factor.

In this report, we investigated whether loss of *TDAG51* alters the development and progression of atherosclerosis by crossing *TDAG51*^*−/−*^ mice^20^ with *ApoE*^*−/−*^ mice, an established hyperlipidemic mouse model of accelerated atherosclerosis.^21^ Our findings provide the first in vivo evidence that deficiency of *TDAG51* reduces atherosclerotic lesion growth. Furthermore, such inhibition of atherogenesis because of *TDAG51* deficiency likely involves the action of PPARγ on specific cellular targets and pathways that are known to affect atherosclerotic lesion development and progression.

## Methods

### Mice and Dietary Conditions

*ApoE*^*−/−*^ mice were obtained from the Jackson Laboratory (Bar Harbor, ME). *TDAG51*‐deficient (*TDAG51*^*−/−*^) mice have been previously described.^20^ *TDAG51*^*−/−*^ (knockout [KO]) mice were backcrossed >9 generations onto a C57BL/6 background. *TDAG51*‐deficient mice were crossbred with *TDAG51*^*+/+*^/*ApoE*^*−/−*^ mice (also on a C57BL/6 background) to generate *TDAG51*^*−/−*^/*ApoE*^*−/−*^ double‐knockout (dKO) mice as well as *ApoE*^*−/−*^ littermate controls. Given previous studies showing that the PPARγ ligand effect on atherosclerosis is sex specific toward male mice,^22^ only male mice were used in this study. Mice were housed with free access to regular chow diet. All experimental procedures using mice were approved by the McMaster University Animal Research Ethics Board.

### Mouse Genotyping Using Polymerase Chain Reaction

Polymerase chain reaction (PCR) was performed to assess the presence of wild‐type (WT) and/or disrupted *TDAG51* alleles using the following primers: WT 1, WT 2, TDAG51 KO 1, and TDAG51 KO 2. PCR‐amplified products (1‐kbp band, wild‐type *TDAG51*; 400‐bp band, disrupted *TDAG51*) were analyzed by agarose gel electrophoresis (AGE). *ApoE* genotyping was confirmed using primers ApoE 1, ApoE 2, and ApoE 3. PCR‐amplified products (155‐bp band, wild‐type *ApoE*; 245‐bp band, disrupted *ApoE*) were analyzed by AGE.

Primers used in this study were WT 1 (5′‐CCG CAG CAC CTC CAA CTC TGC CTG‐3′), WT 2 (5′‐GTC TTC AAA TAC AAT GAA AGA GTC G‐3′), TDAG51 KO 1 (5′‐AAA TGG AAG TAG CAC GTC TCA CTA GTC TCG‐3′), TDAG51 KO 2 (5′‐AGA GCA GCC GAT TGT CTG TTG TGC CCA GTC‐3′), ApoE 1 (5′‐GCC TAG CCG AGG GAG AGC CG‐3′), ApoE 2 (5′‐TGT GAC TTG GGA GCT CTG CAG C‐3′), and ApoE 3 (5′‐GCC GCC CCG ACT GCA TCT‐3′).

### Quantitative RT‐PCR

Gene‐specific primer sets for mouse PPARα, ‐γ, and ‐δ were designed by the Genescript Primer Design Program (http://www.genescript.com). Primer sets for mouse ABCA1 and ABCG1 were purchased from Qiagen (Germantown, MD). Sequences for MCP‐1 and TNFα primers were reported previously.^23^ qRT‐PCR reactions were carried out using SYBR Green, and data was analyzed by the ∆∆C(T) method, normalized to 18s, and shown as fold‐change in expression.

Primers used in this study were MCP‐1 forward primer (5′‐CTC AGC CAG ATG CAG TTA ACG‐3′), MCP‐1 reverse primer (5′‐GGG TCA ACT TCA CAT TCA AAG G‐3′), TNFα forward primer (5′‐TCT CAG CCT CTT CTC ATT CCT‐3′), TNFα reverse primer (5′‐ACT TGG TGG TTT GCT ACG AC‐3′), LXRα forward primer (5′‐GGA GGC AAC ACT TGC ATC CT‐3′), and LXRα reverse primer (5′‐AGG GCT GTA GGC TCT GCT GA‐3′).

### Isolation of Peritoneal Macrophages

Wild‐type C57BL/6 and *TDAG51*^*−/−*^ mice were injected intraperitoneally with 500 μL of 80 μg/mL concanavalin A.^7^ Peritoneal macrophages were harvested 3 days postinjection. Macrophages were cultured in RPMI‐1640 containing 10% FBS and 50 ng/mL macrophage colony stimulating factor.

### Isolation and Culture of Mouse Lung Microvascular Endothelial Cells

Mouse lung microvascular endothelial cells (MLECs) were isolated and cultured using a protocol derived from previous studies^24^ and Miltenyi Biotec (Auburn, CA). Briefly, mouse lung cells were incubated with MACS LSEC (CD146) microbeads (130‐092‐007, Miltenyi Biotec) and subsequently eluted from a MACS Separation LS column (130‐042‐401, Miltenyi Biotec). MLECs were cultured in endothelial cell growth medium (CC‐3121; Lonza, Walkersville, MD).

### Measurement of Total Cholesterol, Triglyceride, and Glucose

Plasma total cholesterol was measured using an Infinity cholesterol measurement kit (Thermo Electron Corporation, Melbourne, Australia). Triglyceride and glucose levels in mice plasma were determined similarly to the above described cholesterol assay (Thermo Electron Corporation).

Macrophages were pretreated in the presence or absence of 10 μmol/L GW9662 in FBS‐deficient media for 4 hour before incubation with acetyl‐LDL (50 μg/mL) or acetyl‐LDL+GW9662 (10 μmol/L) for 24 to 48 hours. Cellular lipids were isolated via Bligh and Dyer chloroform:methanol extraction^25^ with subsequent assessment of total and free cholesterol levels using Cholesterol E and Free Cholesterol E kits (Wako Pure Chemical Industries, Ltd, Osaka, Japan). Lipid content was normalized against cellular protein and the data expressed as fold‐change.

### Measurement of Cholesterol Efflux in Peritoneal Macrophages

HDL/APOA1‐dependent cholesterol efflux to the medium was determined as described previously.^26^ Peritoneal macrophages isolated from *TDAG51*^*−/−*^ and wild‐type C57BL/6 mice were plated at a density of 5×10^5^ cells/well and loaded with 1 μCi/mL [^3^H]cholesterol (PerkinElmer Life Sciences) in RPMI‐1640 media containing 5% LPDS for 48 hours. To equilibrate cholesterol pools, cells were washed twice in media containing 2% fatty acid–free BSA and cultured overnight in the same media. Media were removed, and cells were incubated for 1 to 5 hours in media containing 0.2% BSA in the absence or presence of 40 μg/mL HDL. Following incubation, radioactivity of culture supernatants and cell lysates was measured by liquid scintillation. Results were normalized to total cellular protein content and expressed as the percentage of radioactivity in the medium divided by the total radioactivity in the cells and medium.

### Lactate Dehydrogenase Release Assay

Peritoneal macrophages and MLECs were incubated in the presence or absence of 2.5 μg/mL tunicamycin, 100 nmol/L thapsigargin, or 10 μmol/L 7‐ketocholesterol (7‐KC) for 24 hours. Lactate dehydrogenase release was measured using a Cytotoxicity Detection Kit (Roche, Laval, Canada).

### Detection of Superoxide

Superoxide levels in cells were measured using fluorescent‐dye dihydroethidium (Invitrogen, Carlsbad, CA) as previously described.^16^ Fluorescence intensity is reported as relative fluorescence units (RFU).

### Measurement of Mouse Plasma Lipid Profiles Using FPLC

Mouse plasma was fractionated into lipid components using gel filtration‐fast protein liquid chromatography (FPLC), as described previously.^27^

### Tissue Sample Preparation

Following perfusion–fixation with 10% neutral buffered formalin,^6,28^ hearts (including the aortic roots) were cut transversely and embedded in paraffin. Serial sections, 4 μm thick, were cut starting from the aortic root origin and collected for measurement of lesion size (hematoxylin/eosin staining) and immunohistochemical analyses.^29^ In each mouse, the atherosclerotic lesion area was measured in 5 sections separated by 80 μm (ie, within 320 μm from the aortic valve).^29^ The lesion was traced manually and measured using computer‐assisted image analysis equipment (Olympus BX41 microscope, Olympus DP70 CCD camera, and ImagePro Plus software). Lesion size is expressed as the mean of 5 sections. Therefore, this number is directly proportional to the volume of the lesions in the first 320 μm of the ascending aorta.

### Immunohistochemical Analysis

Immunohistochemical staining of atherosclerotic lesions was performed as described previously.^6,29^ Sections were counterstained with hematoxylin. Human carotid arteries were obtained at the time of endarterectomy from consenting patients. The protocol was approved by the institutional ethics review boards of Hamilton Health Sciences and St. Joseph's Healthcare. Tissue was fixed with formalin and embedded in paraffin. Double immunofluorescence was performed on the sections as described previously.^29^

Antibodies used for immunostaining were anti‐GRP78 (sc‐1050; Santa Cruz, CA), anti‐cleaved caspase‐3 (9661; Cell Signaling, Danvers, MA), anti‐Mac‐3 (55322; BD Pharmingen, San Diego, CA), anti‐KDEL (SPA‐827; Enzo Life Sciences, Farmingdale, NY), anti‐PDI (SPA‐891; Enzo Life Sciences), anti‐PPARγ (07‐466; Upstate, Billerica, MA), anti‐Prdx‐1 (SA‐356; Enzo Life Sciences), anti‐SMA (A2547; Sigma, St. Louis, MO), and anti‐CD3 (A0452; DAKO, Glostrup, Denmark). Nonspecific immunostaining was not detected in control sections. Controls consisted of nonimmune IgG as the primary antibody or the secondary antibody alone. A TACS 2TdT In Situ Apoptosis Detection Kit (Trevigen, Gaithersburg, MD) was used for TUNEL staining. Collagen was stained with Masson's trichrome (HT‐15‐1KT; Sigma).

Immunostaining was quantified by extracting the blue component of the RGB image, thresholding for the staining, and measuring the stained area as well as the total area of the lesion (by manual tracing) using ImagePro 6.3 software. Results are expressed as a percentage of the total lesion area.

### Identification of Insulin and Glucagon in β Cells From Islets of Langerhans

Pancreatic tissue from 25‐week‐old *TDAG51*^*+/+*^/*ApoE*^*−/−*^ or *TDAG51*^*−/−*^/*ApoE*^*−/−*^ mice was immunostained for insulin (red) or glucagon (green). In brief, paraffin sections were deparaffinized and blocked with 5% normal goat serum (Vector, Burlingame, CA). Subsequently, sections were incubated with mouse anti‐insulin antibody cocktail (MS‐1379; Thermo Fisher, Fremont, CA) diluted 1:200, followed by rabbit anti‐glucagon ready‐to‐use antibody (Zymed, San Francisco, CA), for 1 hour each. A mix of goat anti‐rabbit Alexa 488 and goat anti‐mouse Alexa 594 (Molecular Probes, Eugene, OR), diluted 1:200, was applied for 30 minutes. Slides were mounted with Permafluor (Thermo Fisher) and viewed in a Zeiss Axioplan fluorescence microscope.

### Indirect Immunofluorescence of Endogenous TDAG51 Expression in Lesion‐Resident Macrophages

Six‐week‐old *TDAG51*^*+/+*^/*ApoE*^*−/−*^ mice were placed on control chow diet for 25 weeks. Mice were euthanized, and hearts containing the aortic roots were removed, embedded in paraffin, sectioned, and immunostained. Primary antibodies were detected using either Alexa 488 or Alexa 594 donkey anti‐goat IgG (Molecular Probes). A Carl Zeiss LSM510 laser‐scanning confocal microscope was used to examine endogenous TDAG51 localization.

### Collagen Content in Atherosclerotic Lesions From Aortic Roots

Six‐week‐old *TDAG51*^*+/+*^/*ApoE*^*−/−*^ or *TDAG51*^*−/−*^/*ApoE*^*−/−*^ mice were placed on control chow diet for 25 weeks. Hearts containing aortic roots were removed, embedded in paraffin, serially sectioned, and stained with Masson's trichrome. For quantification of collagen content, 1 section per mouse, close to the aortic root origin, was assessed. The amount of collagen was quantified by thresholding for the blue color using Image Pro software, and collagen area is expressed as a percentage of the total lesion area in that section.

### En Face Oil Red O Staining of Mouse Aortas

Unopened aortas were stained with Oil Red O. Aortas were then opened longitudinally, and the stained area was measured as percentage of the total aorta.

### Case–Control Genetic Association Studies

The association between CVD and single‐nucleotide polymorphisms (SNPs) in a 56‐kbp region (chromosome 12, positions 74 680 495 to 74 736 823) that included the entire *TDAG51* gene (chromosome 12, positions 74 705 495 to 74 711 823) and 25 kbp upstream and downstream of the gene was examined in 2 case–control studies. We evaluated results from the CVD case–control study published by the Wellcome Trust Case–Control Consortium (WTCCC).^30^ The WTCCC study included 2000 cases with documented CVD and 3000 controls; all cases and controls were white. The University of California, San Francisco (UCSF) case–control study included 731 myocardial infarction patients and 797 healthy controls, collected by investigators at the UCSF Genomic Resource in Arteriosclerosis.^31^ The number of cases and controls who were successfully genotyped is reported for each genotype group in Tables 3 and 4.

### Statistical Analysis

Experimental values are presented as mean±standard error (SE). Unless otherwise noted, statistical comparisons for all experiments were performed using Mann–Whitney (for 2 groups) and Kruskal–Wallis (for >2 groups) tests. Statistical comparisons for en face Oil Red O staining of mouse aortas and Oil Red O staining of peritoneal macrophages were performed using the unpaired Student *t* test and ANOVA. *P*<0.05 was considered statistically significant for all tests.

The association between SNPs and myocardial infarction was performed by logistic regression analysis that adjusted for age and sex. A combined analysis of the results from the UCSF study and the WTCCC study was carried out using the fixed‐effects Mantel–Haenszel method that combined odds ratios across these studies; homogeneity of the odds ratios was assessed by the Breslow–Day test.

## Results

### Loss of *TDAG51* Reduces Atherosclerotic Lesion Development and Progression

**Figure 1.**
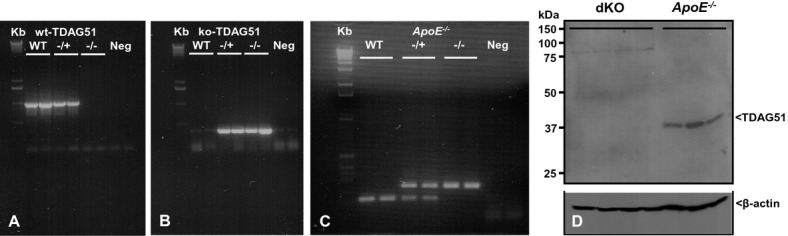
Identification of *TDAG51*^*−/−*^/*ApoE*^*−/−*^ mice. Mouse genomic DNA was amplified by PCR using specific primer sets, and the PCR products were separated on agarose gels to identify mice containing the wild‐type *TDAG51* allele (wt‐TDAG51) (A) or the disrupted *TDAG51* allele (ko‐TDAG51) (B). PCR amplification and gel electrophoresis was also used to identify mice that were deleted for the *ApoE* allele (C). Neg, negative controls for PCR reactions (A through C). Liver tissue obtained from PCR identified *TDAG51*^*−/−*^/*ApoE*^*−/−*^ dKO or control *TDAG51*^*+/+*^/*ApoE*^*−/−*^ mice was homogenized, and total protein lysates were examined by immunoblotting using an anti‐TDAG51 antibody (D). β‐actin was used as a loading control. *TDAG51* indicates T‐cell death‐associated gene 51; PCR, polymerase chain reaction; *ApoE*, apolipoprotein E; ko, knockout; dKO, double knockout.

To investigate the role of *TDAG51* in atherosclerotic lesion development, we generated *TDAG51*^*−/−*^/*ApoE*^*−/−*^ double‐knockout male mice (dKO), as well as *TDAG51*^*+/+*^/*ApoE*^*−/−*^ littermate/sex controls (Figure 1). To focus on the effect of *TDAG51* deficiency, male mice were maintained on a control chow diet, as opposed to a high‐fat chow diet, to minimize the potential contribution of obesity and insulin resistance^32^ to atherosclerotic lesion development and necrosis.

**Figure 2.**
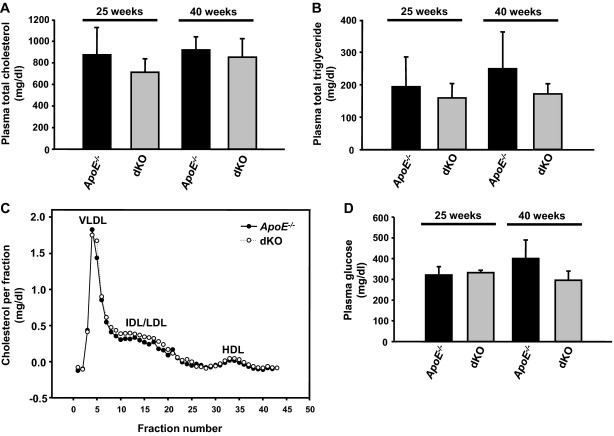
Effect of *TDAG51* deficiency on plasma lipoproteins and glucose in *ApoE*^*−/−*^ mice. *TDAG51*^*−/−*^/*ApoE*^*−/−*^ mice (dKO) and *ApoE*^*−/−*^ control mice were fed standard chow diets for 25 or 40 weeks (n=8 to 9 per group). Following euthanization, plasma was collected, and (A) total cholesterol, (B) triglycerides, (C) lipoprotein profiles, and (D) glucose were determined. Plasma lipoprotein profiles were obtained from 25‐week‐old mice by fast protein liquid chromatography. *TDAG51* indicates T‐cell death‐associated gene 51; *ApoE*, apolipoprotein E; dKO, double knockout; IDL, intermediate‐density lipoprotein; LDL, low‐density lipoprotien; HDL, high‐density lipoprotein.

**Figure 3.**
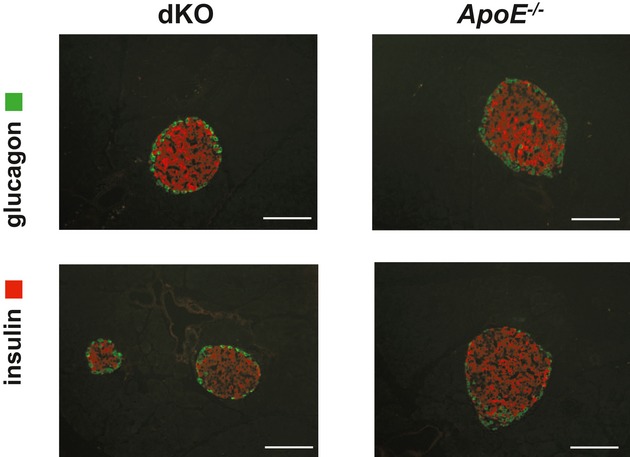
Identification of insulin and glucagon in β cells from the islets of Langerhans. Representative sections from 5 mice per group of pancreatic tissue from 25‐week‐old *TDAG51*^*+/+*^/*ApoE*^*−/−*^ (*ApoE*^*−/−*^) or *TDAG51*^*−/−*^/*ApoE*^*−/−*^ (dKO) mice immunostained for insulin (red) or glucagon (green). Scale bars, 50 μm. *TDAG51* indicates T‐cell death‐associated gene 51; *ApoE*, apolipoprotein E; dKO, double knockout.

Total plasma lipids, glucose, and inflammatory cytokines, as well as aortic atherosclerotic lesion size and composition, were analyzed at 25 and 40 weeks of age. No significant differences in total plasma cholesterol or triglycerides were observed between dKO and *ApoE*^*−/−*^ mice (Figure 2A and 2B). Consistent with these findings, plasma lipid profiles were indistinguishable between these groups at 25 weeks of age (Figure 2C). No significant changes in body weight (data not shown), plasma glucose level (Figure 2D), or morphology of pancreatic β cells from the islets of Langerhans (Figure 3) were observed in dKO and *ApoE*^*−/−*^ mice.

**Figure 4.**
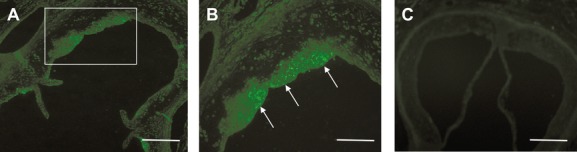
Expression of TDAG51 in atherosclerotic lesions. Six‐week‐old *TDAG51*^*+/+*^/*ApoE*^*−/−*^ and *TDAG51*^*−/−*^/*ApoE*^*−/−*^ mice were fed control chow diet for 25 weeks. Mice were euthanized, and hearts containing aortic roots were removed, embedded in paraffin, sectioned, and immunostained for TDAG51. Indirect immunofluorescence detection of TDAG51 in lesions (arrows) from aortic roots of *TDAG51*^*+/+*^/*ApoE*^*−/−*^ mice (A and B). B, Magnified region from A. Representative images from 5 mice are shown. To assess nonspecific immunofluorescence, aortic root sections from *TDAG51*^*−/−*^/*ApoE*^*−/−*^ mice were immunostained using an anti‐TDAG51 antibody (C). Scale bars: 100 μm in A and C; 50 μm in B. *TDAG51* indicates T‐cell death‐associated gene 51; *ApoE*, apolipoprotein E.

**Figure 5.**
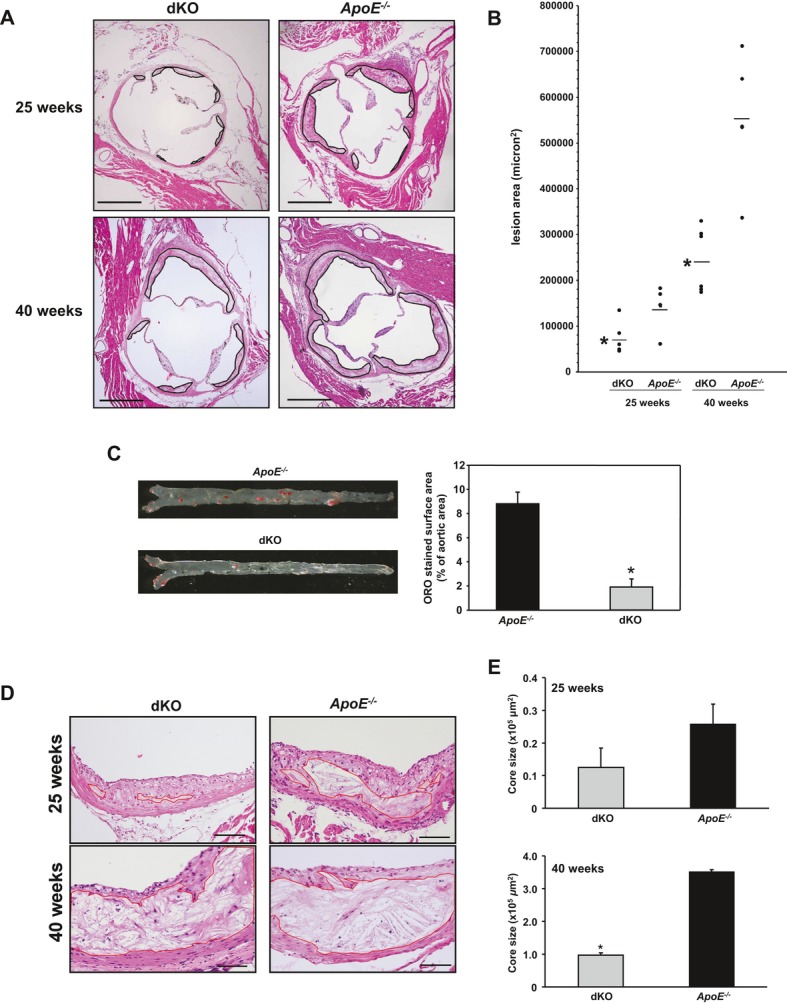
Effect of *TDAG51* deficiency on atherosclerotic lesions in *ApoE*^*−/−*^ mice. *TDAG51*^*−/−*^/*ApoE*^*−/−*^ mice (dKO) and *ApoE*^*−/−*^ control mice were fed standard chow diets for 25 or 40 weeks (n=8 to 9 per group). A and B, Aortic root sections were stained with hematoxylin/eosin, and mean atherosclerotic lesion size was determined. Significant reduction (*P<0.05) in lesion size was observed at both 25 and 40 weeks in dKO compared with *ApoE*^*−/−*^ control groups. Black line demarcates lesion area. Scale bar=500 μm. C, Representative en face Oil Red O (ORO)‐stained aortas and quantitative assessment showed significant reduction (*P=0.0016) in lipid deposition in aortas from 40‐week‐old dKO mice compared with the control group. For quantitative data, results from 3 independent experiments are shown. D, Representative images are shown of necrotic core sizes in aortic root lesions of 25‐ and 40‐week‐old dKO and *ApoE*^*−/−*^ mice (n=5). Red line demarcates necrotic core area. Scale bar=100 μm. E, Necrotic core sizes of 25‐ and 40‐week‐old dKO mice were smaller in aortic root lesions compared with their respective *ApoE*^*−/−*^ control groups (n=5). Data are shown as mean necrotic core area±SE (*P<0.005). *TDAG51* indicates T‐cell death‐associated gene 51; *ApoE*, apolipoprotein E; dKO, double knockout.

As reported previously,^6,15^ TDAG51 is expressed in lesion‐resident macrophages and endothelial cells from 25‐week‐old *ApoE*^*−/−*^ mice (Figure 4). However, this pattern of expression was absent in atherosclerotic lesions from dKO mice. Paraffin sections from the aortic root of dKO and *ApoE*^*−/−*^ mice at 25 or 40 weeks of age were stained with hematoxylin and eosin to assess lesion growth and gross cellular morphology (Figure 5A). Lesion area was reduced by 50% at 25 weeks (0.70±0.14 versus 1.40±0.27 ×10^5^ μm^2^, *P*=0.038) and 56% at 40 weeks (2.45±0.29 versus 5.52±0.63 ×10^5^ μm^2^, *P*=0.0043) (Figure 5B) in dKO mice, compared with *ApoE*^*−/−*^ mice. In addition, en face Oil Red O (ORO) lipid staining (Figure 5C) was decreased by 75% in the aortas of dKO mice at 40 weeks of age (8.9±4.4% versus 2.0±0.9%, *P*<0.005). Although not significant at 25 weeks, mean necrotic core size was reduced by >50% in the dKO mice compared with *ApoE*^*−/−*^ mice (0.12±0.06 versus 0.26±0.06 ×10^5^ μm^2^, *P*=0.26) (Figures 5D and 5E), consistent with the reduction in atherosclerotic lesion size. Normalization to lesion area showed a 14.4±3.8% necrotic core area in the dKO mice, compared to 17.5±2.1% in *ApoE*^*−/−*^ mice at 25 weeks. At 40 weeks mean necrotic core size in dKO mice was significantly smaller than in *ApoE*^*−/−*^ mice (1.0±0.1 versus 3.5±0.6 ×10^5^ μm^2^, *P*<0.005; Figure 5D and 5E), and when normalized to lesion area, necrotic cores of dKO mice were reduced compared with *ApoE*^*−/−*^ controls (41±4% versus 63±8%, *P*<0.05). Thus, in the setting of a normal chow diet, *ApoE*^*−/−*^ mice lacking *TDAG51* exhibited reduced atherosclerotic lesion growth and necrosis.

**Figure 6.**
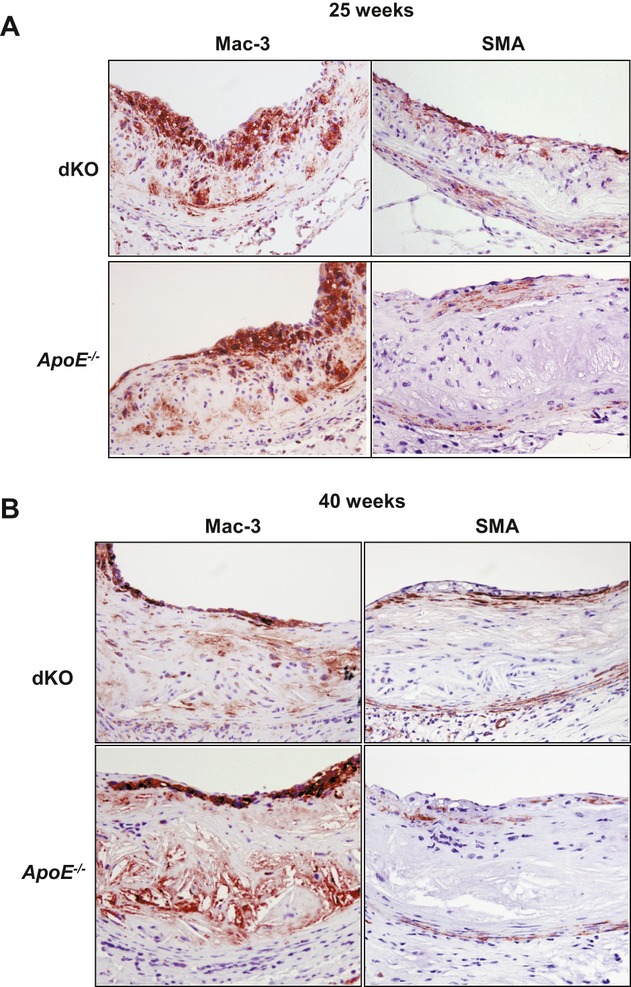
Morphological assessment of atherosclerotic lesions from *TDAG51*^*−/−*^/*ApoE*^*−/−*^ (dKO) and *ApoE*^*−/−*^ control mice. Atherosclerotic lesions from aortic roots of mice fed control chow diet for 25 or 40 weeks (n=8 to 9 per group) were studied. A, Hearts containing aortic roots of 25‐week‐old mice were removed, embedded in paraffin, sectioned, and immunostained for macrophages (Mac‐3) and smooth muscle cells (SMA). A representative image is shown of 5 mice per group. Microscope magnification ×20. B, Hearts containing aortic roots of 40‐week‐old mice were removed, embedded in paraffin, sectioned, and immunostained for Mac‐3 and SMA. A representative image is shown of 5 mice per group. Microscope magnification ×20. *TDAG51* indicates T‐cell death‐associated gene 51; *ApoE*, apolipoprotein E; dKO, double knockout.

**Figure 7.**
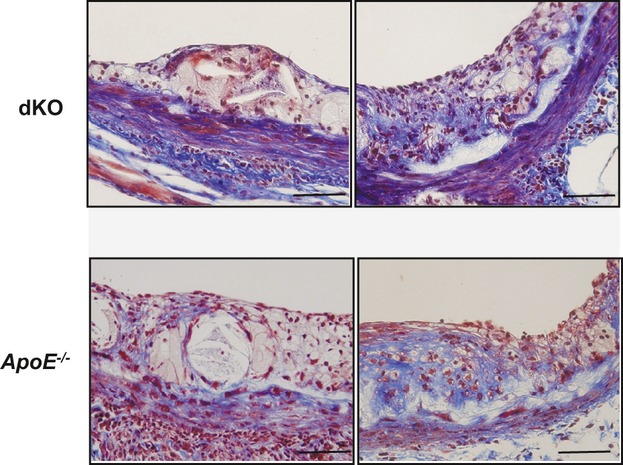
Collagen content in atherosclerotic lesions. Six‐week‐old *TDAG51*^*+/+*^/*ApoE*^*−/−*^ (*ApoE*^*−/−*^) and *TDAG51*^*−/−*^/*ApoE*^*−/−*^ (dKO) mice were fed control chow diet for 25 weeks. Hearts containing aortic roots were removed, embedded in paraffin, sectioned, and stained with Masson's trichrome. Different sections from the same lesion were shown to demonstrate intralesion variability in collagen positivity (blue color). Representative images from 5 mice per group are shown. Scale bar=50 μm. *TDAG51* indicates T‐cell death‐associated gene 51; *ApoE*, apolipoprotein E; dKO, double knockout.

**Figure 8.**
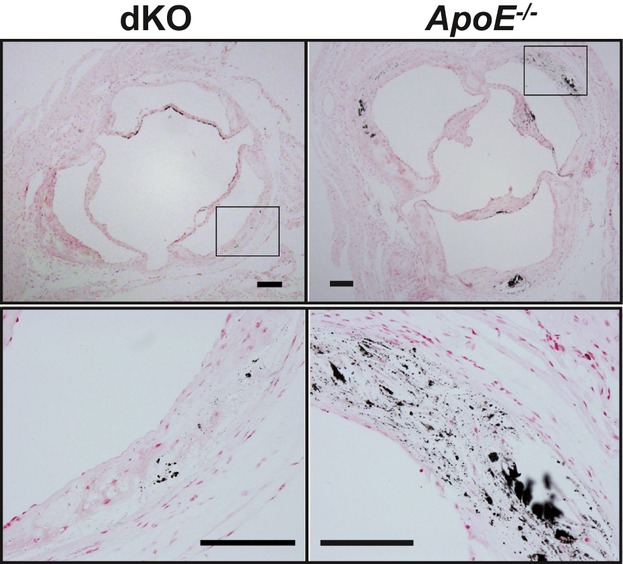
Calcification in atherosclerotic lesions. *TDAG51*^*+/+*^/*ApoE*^*−/−*^ (*ApoE*^*−/−*^) and *TDAG51*^*−/−*^/*ApoE*^*−/−*^ (dKO) mice were fed chow diet for 40 weeks. Hearts containing aortic roots were removed, sectioned, and stained with von Kossa. Representative images from 3 mice per group are shown. Scale bar=100 μm. *TDAG51* indicates T‐cell death‐associated gene 51; *ApoE*, apolipoprotein E; dKO, double knockout.

Immunohistochemical analysis revealed that dKO and *ApoE*^*−/−*^ mice developed fatty streaks and mature atherosclerotic lesions, consisting of macrophages and smooth muscle cells, in the atherosclerotic cap region (Figure 6). However, at 25 and 40 weeks of age, there were no significant differences in the content of either macrophages or smooth muscle cells in the lesions of dKO and *ApoE*^*−/−*^ mice (Figure 6 and Table 1). In support of the en face lipid staining (Figure 5C) and increased necrotic core size (Figure 5D and 5E), cholesterol crystals were prevalent in the necrotic core regions of the 25‐week‐old *ApoE*^*−/−*^ mice (Figure 5D). Collagen content in the lesions, as measured by Masson's trichrome staining, showed high intralesion variability and did not differ significantly between the *ApoE*^*−/−*^ and dKO groups (23.4% versus 27.9%, *P*=0.48; Figure 7). However, at 40 weeks dKO mice exhibited less vascular calcification, as assessed by von Kossa staining, compared with *ApoE*^*−/−*^ mice (Figure 8). Inflammatory cytokines are known to influence several cellular processes that accelerate atherosclerotic lesion growth and stability.^33^ However, loss of *TDAG51* had no significant effect on plasma levels of proinflammatory cytokines, including IL‐12, TNF‐α, or MCP‐1, up to 40 weeks (Table 2). These data suggest the decrease in lesion area was primarily a result of reduction in necrotic core size.

**Table 1.** Lesion Composition of *TDAG51*^*−/−*^/*ApoE*^*−/−*^ and *ApoE*^*−/−*^ Mice

	% Mac‐3 Staining				% SMA Staining			
	dKO	*ApoE* ^−/−^	n	*P*	dKO	*ApoE* ^−/−^	n	*P*
25 Weeks	20.07	5.35	4	NS	3.31	2.38	5	NS
40 Weeks	5.77	3.69	5	NS	0.79	0.42	5	NS

Lesions from 25‐ or 40‐week‐old *TDAG51*^*−/−*^/*ApoE*^*−/−*^ (dKO) and *ApoE*^*−/−*^ mice were sectioned and probed for Mac‐3 (macrophages) and SMA (smooth muscle actin). Macrophage or smooth muscle cell area was measured, and data are expressed as percentage of lesion area. *TDAG51* indicates T‐cell death‐associated gene 51; *ApoE*, apolipoprotein E; dKO, double knockout; n, mouse number; NS, not significant.

**Table 2.** Plasma Inflammatory Marker Levels

	*TDAG51*^*−/−*^/*ApoE*^*−/−*^	*TDAG51*^+/+^/*ApoE*^*−/−*^	*P* Value
15 Weeks			
IL‐12, pg/mL	50.2±18.4 (n=3)	81.3±34.1 (n=3)	NS
TNF‐α, pg/mL	15.3±1.0 (n=3)	16.6±1.8 (n=3)	NS
MCP‐1, pg/mL	258.2±70.2 (n=3)	204±51.2 (n=3)	NS
IFN‐γ, pg/mL	ND	ND	
25 Weeks			
IL‐12, pg/mL	47.5±13.0 (n=5)	60.4±7.4 (n=6)	NS
TNF‐α, pg/mL	19.1±3.7 (n=4)	16.6±1.8 (n=5)	NS
MCP‐1, pg/mL	185.7±51.9 (n=4)	133.9±10.9 (n=5)	NS
IFN‐γ, pg/mL	ND	ND	
40 Weeks			
IL‐12, pg/mL	47.7±10.5 (n=5)	48.7±4.6 (n=6)	NS
TNF‐α, pg/mL	19.0±2.6 (n=6)	18.5±2.6 (n=6)	NS
MCP‐1, pg/mL	157.3±24.3 (n=5)	224±46.9 (n=5)	NS
IFN‐γ, pg/mL	ND	ND	

Data are shown as mean±SE. *TDAG51* indicates T‐cell death‐associated gene 51; *ApoE*, apolipoprotein E; IL‐12, interleukin 12; TNF‐α, tumor necrosis factor α; MCP‐1, monocyte chemoattractant protein‐1; IFN‐γ, interferon γ; NS, not significant vs age‐matched groups; ND, not detected.

### *TDAG51* Deficiency Is Cytoprotective Against ER Stress and Oxidative Stress

**Figure 9.**
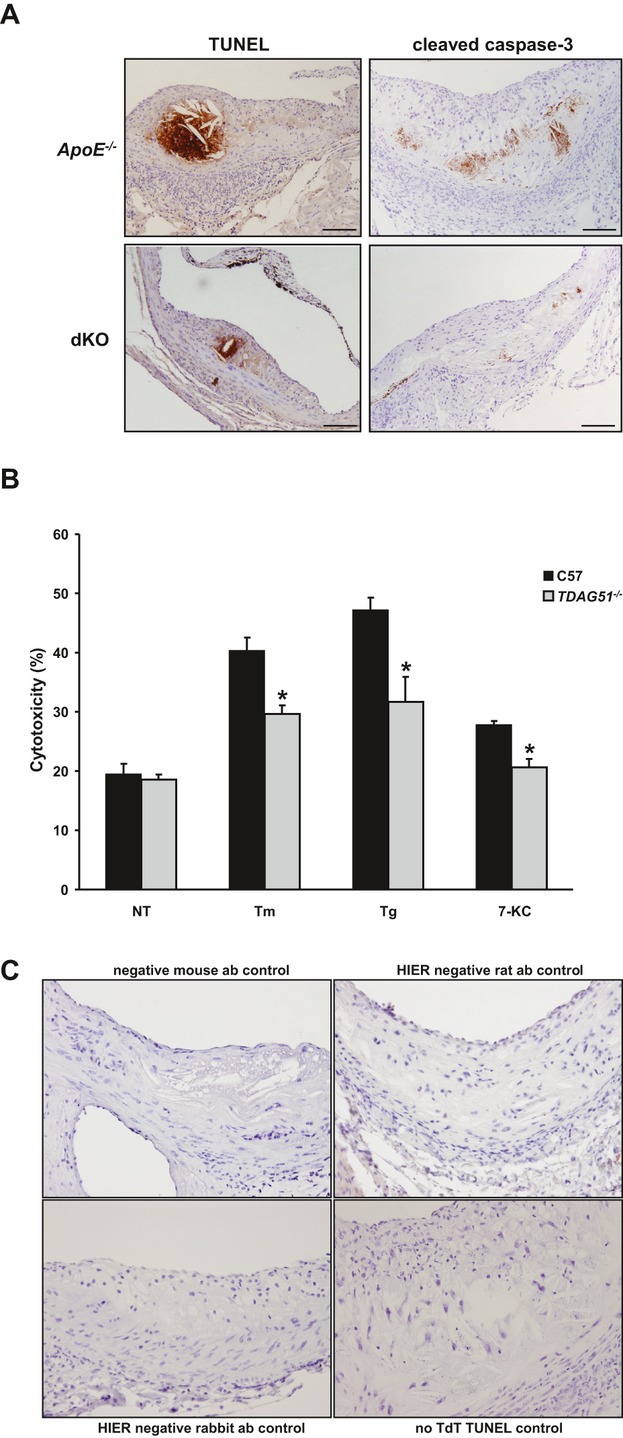
*TDAG51* deficiency reduces cell death in atherosclerotic lesions and peritoneal macrophages. *TDAG51*^*−/−*^/*ApoE*^*−/−*^ (dKO) or *ApoE*^*−/−*^ mice were placed on control chow diet for 25 weeks. A, Representative images of atherosclerotic lesions from 5 mice per group were stained for TUNEL and cleaved caspase‐3. Microscope magnification ×20. B, Peritoneal macrophages isolated from wild‐type C57BL/6 (C57) mice or *TDAG51*^*−/−*^ mice were treated with 2.5 μg/mL tunicamycin (Tm), 100 nmol/L thapsigargin (Tg), or 10 μmol/L 7‐ketocholesterol (7‐KC) for 24 hours. Cytotoxicity was determined by measuring LDH release. Mean±SE from 5 independent experiments are shown. *P<0.05 relative to C57 controls. C, Negative controls for IHC sections. Primary antibodies were omitted, and only secondary antibodies were used (anti‐mouse, anti‐rat, or anti‐rabbit, with heat‐induced epitope retrieval [HIER] where specified). Negative control for the TUNEL staining had no terminal deoxynucleotidyl transferase (TdT) added to the staining mixture. *TDAG51* indicates T‐cell death‐associated gene 51; *ApoE*, apolipoprotein E; dKO, double knockout; LDH, lactate dehydrogenase; IHC, immunohistochemistry; PPARγ, peroxisome proliferator‐activated receptor γ; ab, antibody; NT, nontreated.

*TDAG51* expression is increased by ER stress and causes detachment‐induced apoptotic cell death when overexpressed.^15,34^ Recent studies have reported that reducing ER stress by treatment with the small chemical chaperone 4‐PBA^35^ or by genetic ablation of CHOP^5^ attenuates apoptosis, plaque necrosis, and atherogenesis in *ApoE*^*−/−*^ mice. To investigate whether the absence of *TDAG51* decreases apoptosis in vivo, atherosclerotic lesions were stained for TUNEL and activated caspase‐3.^6^ TUNEL staining as well as activated caspase‐3 staining was reduced in the necrotic core of advanced lesions from dKO mice, compared with *ApoE*^*−/−*^ mice (Figure 9A). In support of these findings, we observed that *TDAG51*^*−/−*^ peritoneal macrophages (Figure 9B) were resistant to cell death induced by ER stress and oxidative stress.

### *TDAG51* Negatively Regulates the Expression of *PPARγ* and Its Downstream Targets

**Figure 10.**
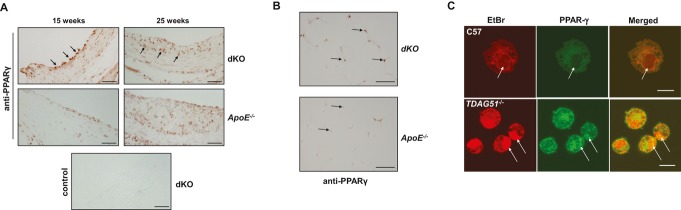
*TDAG51* deficiency increases PPARγ expression and nuclear localization in lesion‐resident macrophages. A, *TDAG51*^*−/−*^/*ApoE*^*−/−*^ (dKO) or *ApoE*^*−/−*^ mice were placed on control chow diet for 15 or 25 weeks. Atherosclerotic lesions from the aortic roots were sectioned and immunostained for PPARγ. Arrows indicate PPARγ‐positive staining macrophages. Representative images from 5 mice per group are shown. Scale bar=50 μm. B, Identification of PPARγ in adipose tissue from *TDAG51*^*+/+*^/*ApoE*^*−/−*^ (*ApoE*^*−/−*^) and *TDAG51*^*−/−*^/*ApoE*^*−/−*^ (dKO) mice fed chow diet for 40 weeks. Fat pads were removed, embedded in paraffin, sectioned, and immunostained for PPARγ. Arrows indicate positive nuclear immunostaining for PPARγ. Consistent with lesion‐resident *TDAG51*^*−/−*^ macrophages, intensity of nuclear PPARγ staining was increased in *TDAG51*^*−/−*^ adipocytes. Representative images from 5 mice per group are shown. Scale bar=100 μm. C, Optical sections 0.8 μm in thickness were obtained through *TDAG51*^*−/−*^ and C57BL/6 (C57) peritoneal macrophages at the plane of the nuclei (arrows). In C57 macrophages, little PPARγ (green) was visualized within the nucleus, whereas in *TDAG51*^*−/−*^ macrophages, PPARγ (green) was found to colocalize (merged green and red producing yellow) with nucleic acids (red) as shown by ethidium bromide (EtBr) staining in the nuclei (arrows). Representative images from 3 independent experiments are shown. Scale bar=10 μm. *TDAG51* indicates T‐cell death‐associated gene 51; *ApoE*, apolipoprotein E; dKO, double knockout; PPARγ, peroxisome proliferator‐activated receptor γ.

**Figure 11.**
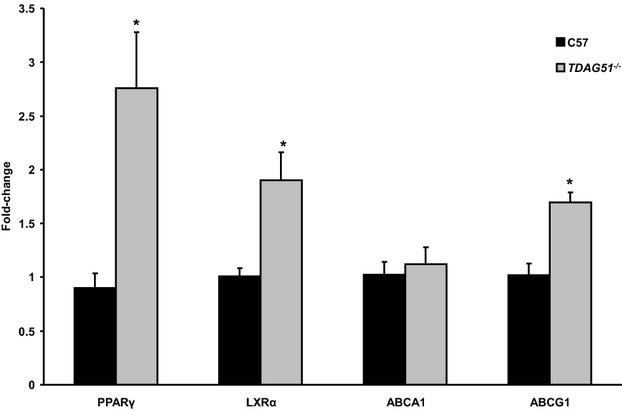
Detection of PPARγ and its target genes in peritoneal macrophages. Total RNA was isolated from male *TDAG51*^*−/−*^ or C57BL/6 (C57) peritoneal macrophages and mRNA expression assessed by qRT‐PCR. Data were normalized to 18s, and fold‐change in expression relative to C57 was determined. Results from 5 independent experiments are shown as mean±SE. *P<0.05, relative to C57 controls. PPARγ indicates peroxisome proliferator‐activated receptor γ; *TDAG51*, T‐cell death‐associated gene 51; qRT‐PCR, quantitative real‐time polymerase chain reaction.

To provide further mechanistic insight into the antiatherogenic effects of *TDAG51* deficiency, we investigated the expression of *PPARγ* because of its involvement in modulating various pathways contributing to atherogenesis, such as inflammation, lipid metabolism, and oxidative stress. Furthermore, we have previously observed an inverse correlation between *TDAG51* and *PPARγ* expression in 3T3‐L1 cells.^18^ Prior studies have demonstrated the expression of *PPARγ* in lesion‐resident macrophages from *LDLR*^*−/−*^ mice.^36^ To determine whether deficiency of *TDAG51* alters the expression of *PPARγ* in lesional macrophages, paraffin sections from the aortic roots of chow‐fed dKO and *ApoE*^*−/−*^ mice after 15 or 25 weeks were immunostained for PPARγ (Figure 10A). Intense nuclear staining for PPARγ was observed in lesion‐resident macrophages from dKO mice compared with *ApoE*^*−/−*^ mice. As a positive control, nuclear staining for PPARγ was observed in adipocytes from dKO and *ApoE*^*−/−*^ mice (Figure 10B). Again, the intensity of nuclear staining for PPARγ was increased in adipocytes from dKO mice as well as in cultured *TDAG51*^*−/−*^ macrophages (Figure 10C). Furthermore, *TDAG51*^*−/−*^ peritoneal macrophages exhibited increased mRNA expression of *PPARγ* (2.8±0.5‐fold, *P*<0.05) and its target gene, *LXRα* (1.9±0.3‐fold, *P*<0.05), as well as PPARγ‐inducible gene *ABCG1* (1.7±0.1‐fold, *P*<0.05), but not *ABCA1,* compared with wild‐type macrophages (Figure 11).

Consistent with these findings, reintroduction of *TDAG51* using a retrovirus expression construct caused a significant reduction in *PPARγ* mRNA expression (*P*<0.001) in *TDAG51*^*−/−*^ mouse embryonic fibroblasts (data not shown). Furthermore, siRNA knockdown of *TDAG51* in HeLa cells also showed enhanced *PPARγ* expression (data not shown).

### *TDAG51* Deficiency Enhances PPARγ‐Dependent Inhibition of Inflammatory Cytokine Expression in Macrophages

**Figure 12.**
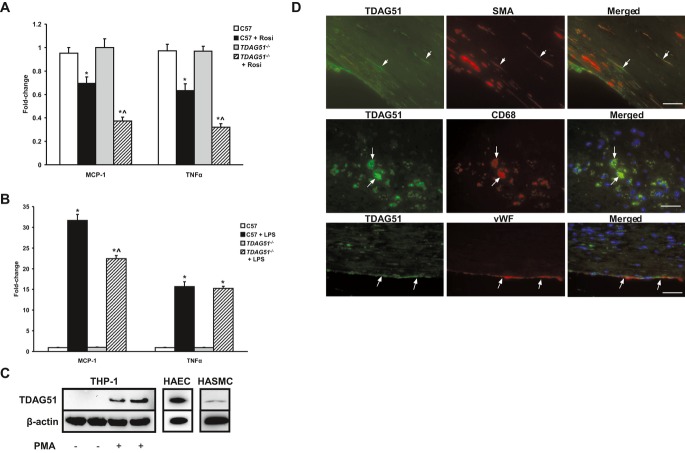
*TDAG51* deficiency increases rosiglitazone‐dependent inhibition of inflammatory marker expression in peritoneal macrophages. A, Peritoneal macrophages isolated from *TDAG51*^*−/−*^ or C57BL/6 (C57) mice were incubated in the presence or absence of 20 μmol/L rosiglitazone (Rosi). mRNA expression of MCP‐1 and TNF‐α were assessed by qRT‐PCR. Data from 6 independent experiments are shown as mean±SE. *P<0.05 vs nontreated controls; ^*P*<0.05 vs C57+rosiglitazone. B, Peritoneal macrophages isolated from *TDAG51*^*−/−*^ or C57BL/6 (C57) mice were incubated in the presence or absence of 50 ng/mL lipopolysaccharide (LPS). mRNA expression of MCP‐1 and TNF‐α was assessed by qRT‐PCR. Data from 6 independent experiments are shown as mean±SE. *P<0.05 vs controls; ^*P*<0.05 vs C57+LPS. C, THP‐1 monocytes were incubated in the presence or absence of 100 nmol/L PMA for 48 hours. Following incubation, THP‐1 monocytes and PMA‐derived THP‐1 macrophages were immunoblotted for TDAG51. Untreated human aortic endothelial cells (HAECs) and human aortic smooth muscle cells (HASMCs) were also immunoblotted for TDAG51. β*‐*actin was used as a loading control. Representative immunoblots from 3 independent experiments are shown. D, Atherosclerotic lesions from human carotid arteries were stained by double immunofluorescence to identify the cell types positive for TDAG51. Some of the smooth muscle cells (positive for smooth muscle actin [SMA]), macrophages (positive for CD68), and endothelial cells (positive for vWF) were also positive for TDAG51 (arrows). Scale bar=100 μm. *TDAG51* indicates T‐cell death‐associated gene 51; qRT‐PCR, quantitative real‐time polymerase chain reaction; TNF‐α, tumor necrosis factor α; vWF, von Willebrand factor; MCP‐1, monocyte chemoattractant protein‐1; THP‐1, human monocytic cell line; PMA, phorbol 12‐myristate 13‐acetate.

Inflammation has been shown to modulate several processes that contribute to atherogenesis and lesion stability.^33^ *TDAG51*^*−/−*^ peritoneal macrophages exhibited no basal differences in the expression of either MCP‐1 or TNFα relative to controls, as assessed by RT‐PCR (Figure 12A). However, in the presence of the PPARγ agonist rosiglitazone, *TDAG51*^*−/−*^ macrophages expressed significantly lower levels of both MCP‐1 and TNFα, relative to wild‐type macrophages (Figure 12A). Furthermore, *TDAG51* deficiency caused a significant reduction in LPS‐induced expression of MCP‐1 (22.4±0.8‐fold versus 31.6±1.5‐fold, *P*<0.05), but not TNF‐α, compared with wild‐type macrophages (Figure 12B). Human THP‐1‐derived macrophages, but not THP‐1 monocytes, as well as aortic endothelial cells (HAECs) and aortic smooth muscle cells (HASMCs), expressed TDAG51 protein (Figure 12C). Consistent with these findings, TDAG51 was also expressed in macrophages (CD68), smooth muscle cells (SMA), and endothelial cells (vWF) in lesions from human carotid arteries, as determined by dual immunohistochemistry (Figure 12D).

### Loss of TDAG51 Increases Cholesterol Efflux in Cultured Peritoneal Macrophages

**Figure 13.**
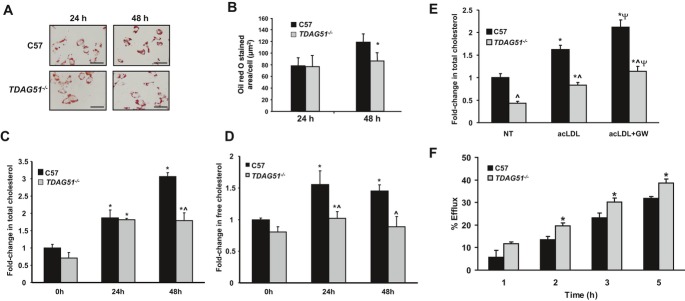
Lipid accumulation and cholesterol efflux in *TDAG51*^*−/−*^ peritoneal macrophages. A, Representative images from 3 independent experiments of Oil Red O (ORO) staining of C57BL/6 (C57) or *TDAG51*^*−/−*^ macrophages treated with acetylated LDL for 24 or 48 hours. Scale bar=20 μm. The amount of ORO staining was quantified B and is shown as mean±SE from 3 independent experiments. *P<0.05 compared with C57 macrophages. C and D, C57BL/6 (C57) or *TDAG51*^*−/−*^ peritoneal macrophages were incubated in the presence or absence of 50 μg/mL acetylated LDL for 24 or 48 hours. Lipids were extracted and cellular total C or free D cholesterol assessed biochemically. Cholesterol was normalized to cellular protein, and fold‐change relative to C57 controls was calculated. *P<0.05 vs 0 hours controls; ^*P*<0.05 vs C57 at the same point. Data are shown as mean fold‐change±SE (n=6). E, C57BL/6 (C57) or *TDAG51*^*−/−*^ peritoneal macrophages were incubated in the presence or absence of 50 μg/mL acetylated LDL or 10 μmol/L GW9662 for 48 hours. Lipids were extracted and cellular total cholesterol assessed. *P<0.05 vs nontreated (NT) controls; ^*P*<0.05 vs C57 controls; ^Ψ^*P*<0.05 vs acLDL‐treated groups. Data are shown as mean fold‐change±SE (n=10). F, HDL‐dependent cholesterol efflux from *TDAG51*^*−/−*^ or C57 peritoneal macrophages. Mean±SE from 5 independent experiments is shown. *P<0.05 compared with C57 controls. *TDAG51* indicates T‐cell death‐associated gene 51; LDL, low‐density lipoprotein; HDL, high‐density lipoprotein; acLDL, acetyl‐LDL; acLDL+GW, acetyl‐LDL+GW9662.

Given that PPARγ increases reverse cholesterol transport in lesion‐resident macrophages and is considered atheroprotective,^37–38^ we examined the effects of *TDAG51* deficiency on macrophage foam cell formation and cholesterol efflux. To assess macrophage foam cell formation, peritoneal macrophages were cultured in the absence or presence of acetylated low‐density lipoprotein (LDL), and the accumulation of intracellular lipids was examined by ORO staining (Figure 13A). *TDAG51*^***−/−***^ macrophages showed a significant reduction in ORO staining at 48 hours compared with wild‐type macrophages (Figure 13B). *TDAG51*^***−/−***^ macrophages also accumulated significantly less cellular total cholesterol (1.8±0.1‐fold versus 3.1±0.1‐fold, *P*<0.05; Figure 13C) and free cholesterol (0.9±0.1‐fold versus 1.5±0.1‐fold, *P*<0.05; Figure 13D) after incubation with acetyl‐LDL (50 μg/mL) for 48 hours, relative to wild‐type macrophages. Consistent with the hypothesis that increased PPARγ results in reduced lipid accumulation, the PPARγ antagonist GW9662 (10 μmol/L) increased cellular total cholesterol in both wild‐type and KO macrophages after 48 hours incubation with 50 μg/mL acetyl‐LDL, compared with acetyl‐LDL treatment alone (Figure 13E). To determine whether the reduced lipid accumulation with *TDAG51* deficiency was the result of increased cholesterol efflux, macrophages were loaded with [^3^H]cholesterol, and the percent change in intracellular radiolabeled cholesterol in the presence or absence of high‐density lipoprotein (HDL) was measured (Figure 13F). Cholesterol efflux was significantly increased in *TDAG51*^***−/−***^ peritoneal macrophages 2, 3, and 5 hours after the addition of HDL, compared with WT macrophages (Figure 13F). PPARγ agonists induce the expression of ABCG1, a protein transporter mediating cholesterol efflux from macrophages.^39^ Consistent with these results, *ABCG1* mRNA expression, but not *ABCA1* expression, was significantly increased in *TDAG51*^***−/−***^ peritoneal macrophages compared with wild‐type macrophages (Figure 11). Taken together, these results suggest the cumulative effect of increased cholesterol efflux over time leads to significantly lower intracellular total and free cholesterol levels in *TDAG51*^***−/−***^ macrophages.

### Effect of *TDAG51* Deficiency on Peroxiredoxin 1 Expression

**Figure 14.**
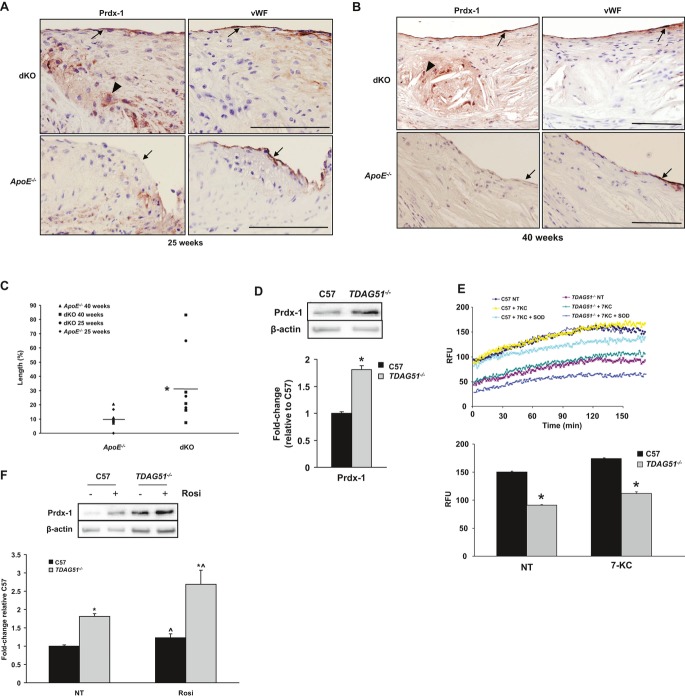
*TDAG51* deficiency increases Prdx‐1 expression in atherosclerotic lesions and reduces intracellular superoxide levels in *TDAG51*^*−/−*^ peritoneal macrophages. *TDAG51*^*−/−*^/*ApoE*^*−/−*^ (dKO) or *ApoE*^*−/−*^ mice were placed on control chow diet. Atherosclerotic lesions from aortic roots of (A) 25‐week‐old mice or (B) 40‐week‐old mice were sectioned and consecutive sections immunostained for Prdx‐1 and von Willebrand factor (vWF). Arrows indicate endothelium; arrowheads indicate macrophages/foam cells. Scale bar=100 μm. Representative images are shown from 5 mice per group. C, Percentage of Prdx‐1 positivity in endothelium (measured as the length of Prdx1‐positive endothelium divided by the total length of endothelium as immunostained with vWF) of 25‐ and 40‐week‐old dKO and *ApoE*^*−/−*^ mice. The averages of 5 sections per mouse were assessed. *P<0.05 compared with *ApoE*^*−/−*^ (n=8 to 9 for each genotype). D, Peritoneal macrophages isolated from *TDAG51*^*−/−*^ mice exhibited elevated levels of Prdx‐1 protein, as determined by immunoblotting. Data are shown as mean±SE (n=9). *P<0.05 relative to C57BL/6 (C57) macrophages. E, *TDAG51*^*−/−*^ peritoneal macrophages displayed lower levels of superoxide at both baseline and when subjected to 10 μmol/L 7‐ketocholesterol (7‐KC). Mean±SE from 6 independent experiments is shown. *P<0.05 relative to C57 macrophages. F, Peritoneal macrophages from C57BL/6 or *TDAG51*^*−/−*^ mice were incubated in the presence or absence of 20 μmol/L rosiglitazone (Rosi) for 18 hours and then immunoblotted for Prdx‐1. Data are shown as mean±SE (n=9). *P<0.05 vs C57 controls; ^*P*<0.05 vs respective nontreated (NT) macrophages. *TDAG51* indicates T‐cell death‐associated gene 51; Prdx‐1, peroxiredoxin‐1; *ApoE*, apolipoprotein E; dKO, double knockout; RFU, relative fluorescence unit.

**Figure 15.**
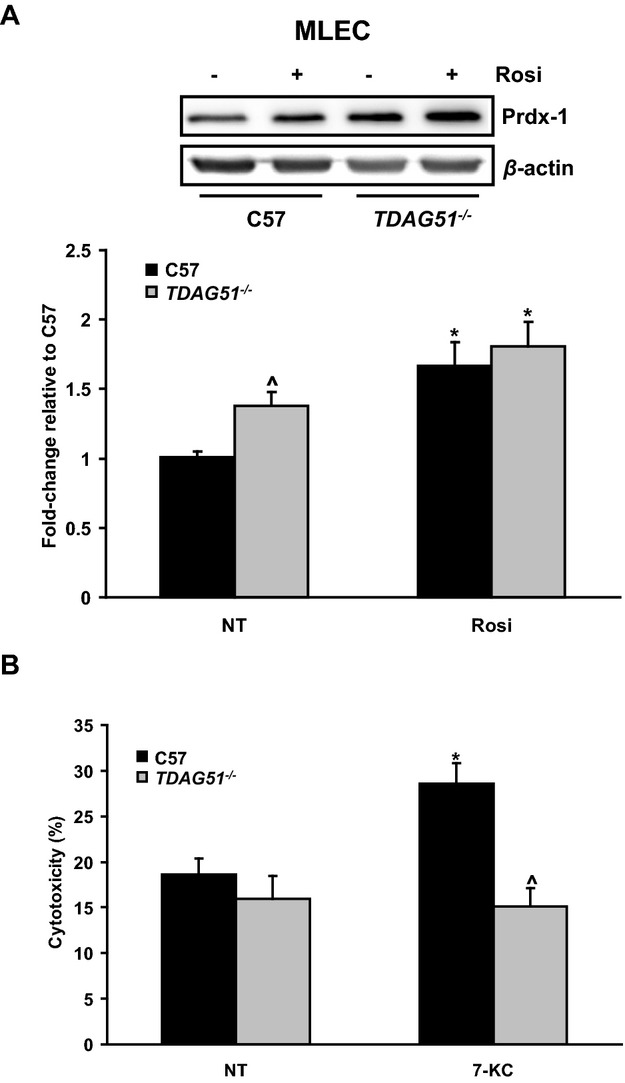
Prdx‐1 expression in *TDAG51*^*−/−*^ mouse lung endothelial cells. A, Mouse lung endothelial cells (MLECs) from C57BL/6 (C57) or *TDAG51*^*−/−*^ mice were treated in the presence or absence of 20 μmol/L rosiglitazone (Rosi) for 18 hours. After incubation, Prdx‐1 protein levels were assessed by immunoblotting. Data are shown as mean±SE (n=10). *P<0.05 vs respective nontreated (NT) controls; ^*P*<0.05 vs nontreated C57. B, C57BL/6 and *TDAG51*^*−/−*^ mouse lung endothelial cells were incubated in the presence or absence of 5 μmol/L 7‐ketocholesterol (7‐KC) for 24 hours. Cytotoxicity was determined by measuring LDH release. Data are shown as mean±SE (n=7). *P<0.05 vs nontreated C57; ^*P*<0.05 vs 7‐KC‐treated C57. *TDAG51* indicates T‐cell death‐associated gene 51; Prdx‐1, peroxiredoxin‐1; *ApoE*, apolipoprotein E; LDH, lactate dehydrogenase.

**Figure 16.**
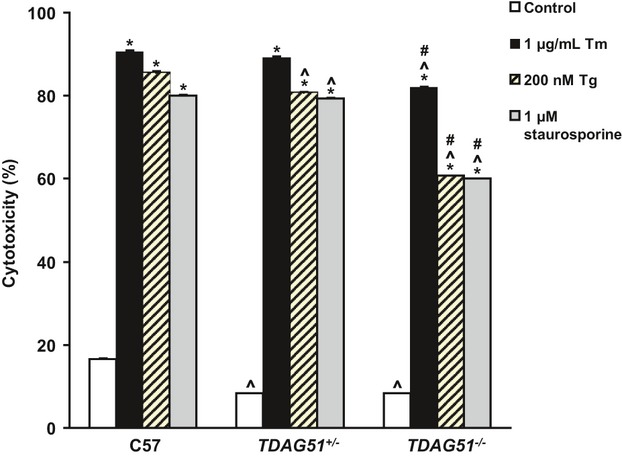
*TDAG51* deficiency protects vascular smooth muscle cells (VSMCs) against endoplasmic reticulum (ER) and oxidative stress. VSMCs isolated from C57BL/6 (C57), *TDAG51*^*+/−*^, and *TDAG51*^*−/−*^ mice were incubated in the presence or absence of 1 μg/mL tunicamycin (Tm), 200 nmol/L thapsigargin (Tg), or 1 μmol/L staurosporine for 48 hours. Cytotoxicity was determined by measuring LDH release. Results from 4 independent experiments are shown as mean±SE. *P<0.05 relative to respective genotype controls; ^*P*<0.05 compared with respective C57 group; ^#^*P*<0.05 compared with respective *TDAG51*^*+/−*^ group. *TDAG51* indicates T‐cell death‐associated gene 51; LDH, lactate dehydrogenase.

Recent studies have suggested that loss of peroxiredoxin 1 (Prdx‐1), a member of a ubiquitous family of antioxidant enzymes,^40^ reduces endothelial cell activation and early atherosclerosis.^41^ Because an increase in Prdx‐1 expression could contribute to the observed cytoprotective effect of *TDAG51* deficiency against oxidative stress (Figure 9B), lesions from dKO and *ApoE*^*−/−*^ mice were immunostained for Prdx‐1. At both 25 (36.4% versus 8.4%, *P*=0.10) and 40 (31.3% versus 11.2%, *P*=0.15) weeks, the percentage of Prdx‐1 immunopositivity in the endothelium was increased in dKO mice compared with *ApoE*^*−/−*^ controls (Figure 14A and 14B). Furthermore, when mice from 25 and 40 weeks were combined, dKO mice exhibited significantly more Prdx‐1 immunopositivity than *ApoE*^*−/−*^ mice (33.2% versus 10.1%, *P*=0.015; Figure 14C). In addition, *TDAG51*^*−/−*^ peritoneal macrophages (Figure 14D) as well as lung microvascular endothelial cells (Figure 15A) exhibited increased Prdx‐1 protein expression. This correlated with a significant decrease in superoxide levels at basal conditions and improved resistance to 7‐ketocholesterol‐induced oxidative stress (Figures 9B, 14E, and 15B). A significant increase in Prdx‐1 protein was also observed in *TDAG51*^*−/−*^ peritoneal macrophages (2.7±0.4‐fold, *P*<0.05) following treatment with the PPARγ agonist rosiglitazone, compared with wild‐type cells (1.2±0.1‐fold, *P*<0.05; Figure 14F). Rosiglitazone treatment also induced Prdx‐1 protein expression in microvascular endothelial cells, although no significant differences were observed between *TDAG51*^*−/−*^ and wild‐type cells (Figure 15A). Although *TDAG51*^*−/−*^ mouse aortic smooth muscle cells also exhibited increased resistance to oxidative stress‐induced cell death in vitro (Figure 16), no differences in lesion smooth muscle cell apoptosis were observed in vivo (Figure 9A).

### Single‐Nucleotide Polymorphisms in the Human *TDAG51* Gene Are Associated With CVD

The WTCCC case–control study of CVD published data for 5 single‐nucleotide polymorphisms (SNPs) in the 56‐kbp region that included the *TDAG51* gene.^30^ Two of these SNPs (rs10880022 and rs2367446) were associated with CVD in this study (Table 3); both remained significant after Bonferroni correction for testing 5 SNPs. We genotyped rs10880022 and rs2367446 in the UCSF study^31^ and found that rs2367446 was also associated with myocardial infarction (OR, 1.24; 95% CI, 1.04 to 1.48; *P*=0.019). A combined analysis of rs2367446 in both studies found that it was associated with CVD, with an odds ratio of 1.15 (95% CI, 1.07 to 1.24, *P=*0.00031; Table 4); this *P* value remained significant after Bonferroni correction for testing 5 SNPs.

**Table 3.** Association of SNPs in the *TDAG51* Region With Myocardial Infarction in the WTCCC Study

SNP	Chromosome	Position	Case		Control		*P* Value (Additive)*
			Count	MAF	Count	MAF	
rs10880021	12	74 684 730	1823	0.150	2824	0.143	0.31
rs10880022	12	74 684 852	1879	0.406	2875	0.434	0.0084
rs2367446	12	74 685 000	1850	0.372	2811	0.343	0.0047
rs17835106	12	74 692 068	1923	0.171	2933	0.181	0.20
rs7976730	12	74 734 851	1924	0.376	2936	0.376	0.97

SNP indicates single‐nucleotide polymorphism; *TDAG51*, T‐cell death‐associated gene 51; WTCCC, Wellcome Trust Case–Control Consortium; MAF, minor allele frequency.

* *P* value is from multivariable logistic regression model.

**Table 4.** Association of rs2367446 With CVD

Study	Case				Control				OR (95% CI)*	*P* Value*
	CC	CT	TT	MAF	CC	CT	TT	MAF		
UCSF	93	321	309	0.351	76	333	373	0.310	1.24 (1.04 to 1.48)	0.019
WTCCC	248	880	722	0.372	325	1281	1205	0.343	1.13 (1.04 to 1.23)	0.005
Combined	341	1201	1031	0.366	401	1614	1578	0.336	1.15 (1.07 to 1.24)	3.1×10^−^^4^

CVD indicates cardiovascular disease; UCSF, University of California, San Francisco; WTCCC, Wellcome Trust Case–Control Consortium; MAF, minor allele frequency; OR, odds ratio; CI, confidence interval. CC, CT, TT represent genotype.

* *P* value and OR are from multivariable logistic regression model of UCSF and WTCCC and from Mantel–Haenszel for the combined studies.

## Discussion

Several lines of evidence suggest that *TDAG51* modulates lesion progression and plaque stability. First, TDAG51 is expressed in athero‐prone vascular endothelial cells^19^ as well as lesion‐resident macrophages and endothelial cells from *ApoE*^*−/−*^ mice with diet‐induced hyperhomocysteinemia.^6,15^ Second, TDAG51 is expressed in apoptotic cells within the lipid‐rich necrotic core.^6,15^ Third, overexpression of TDAG51 in cultured human vascular endothelial cells leads to detachment‐induced apoptosis.^15,34^ Additional observations of increased TDAG51 expression during all stages of atherogenesis in the absence of HHcy^6^ suggest that other pathophysiological conditions that induce ER stress and/or TDAG51 expression modulate lesion growth and stability. In support of this hypothesis, we have reported that peroxynitrite, a proatherogenic agent generated from nitric oxide and superoxide, induces ER stress, TDAG51 expression, and apoptosis in cultured vascular endothelial cells.^16^ Given that ER stress plays a major role in lesion progression and plaque stability^5–7,5–43^ and that *TDAG51* is an ER stress‐inducible gene^6,14–16^ expressed in lesion resident macrophages and endothelial cells, we sought to investigate the causal role of *TDAG51* in atherogenesis.

In this study, *TDAG51*^*−/−*^/*ApoE*^*−/−*^ dKO male mice showed significant reductions in lesion and necrotic lipid core sizes in aortic roots at 25 and 40 weeks compared with age‐matched *ApoE*^*−/−*^ controls. Thus, in the setting of a normal chow diet, *ApoE*^*−/−*^ mice lacking *TDAG51* exhibited reduced growth of atherosclerotic lesions.

As TDAG51 is expressed in lesion‐resident cells undergoing apoptotic cell death,^6,15^ a potential mechanism through which *TDAG51* deficiency contributes to the reduced atherosclerotic lesion size and necrosis observed in this study is decreased lesional apoptosis. Decreased lesional necrosis from dKO mice was associated with reduced apoptosis, as determined by immunohistochemical staining for TUNEL and cleaved caspase‐3. Our data are consistent with the hypothesis that retardation of the rate of necrotic core and lesion growth is in part a consequence of increased resistance to macrophage cell death resulting from *TDAG51* deficiency. Consistent with this hypothesis, peritoneal macrophages isolated from *TDAG51*^*−/−*^ mice were resistant to ER and oxidative stress‐induced cell death. Cytoprotection was associated with increased levels of Prdx‐1 in lesions of dKO mice as well as in *TDAG51*^*−/−*^ macrophages and endothelial cells. This was accompanied by reduced superoxide levels in *TDAG51*^*−/−*^ macrophages at both baseline and when exposed to oxidative stress, a finding consistent with previous studies.^44–45^ These data suggest that the elevation of Prdx‐1 levels associated with *TDAG51* deficiency contributes to increased resistance to oxidative stress‐induced apoptosis, thereby reducing atherosclerotic lesion progression and rupture. This is consistent with the finding that *Prdx‐1* deficiency in *ApoE*^*−/−*^ mice causes endothelial activation (increased leukocyte rolling, endothelial P‐selectin, and vWF expression) and accelerates atherosclerosis.^41^ Further studies should clarify whether TDAG51‐mediated endothelial dysfunction contributes to atherogenesis.

It has been previously reported that inhibiting macrophage apoptosis increases atherosclerotic lesion size. Macrophage‐specific deletion of the proapoptotic gene *p53* resulted in significantly increased lesion area after 15 or 20 weeks on a high‐fat diet in *LDLR*^*−/−*^ mice.^46^ However, the authors attributed the larger lesion size to increased cell proliferation rather than decreased apoptosis, as no significant differences in apoptosis were observed. Liu et al^11^ demonstrated that macrophage‐specific deletion of Bax, a proapoptotic protein, decreased macrophage apoptosis and was associated with increased lesion area in *LDLR*^*−/−*^ mice after 10 weeks on a Western diet. Alternatively, deletion of the macrophage apoptosis inhibitory factor (AIM) was associated with increased apoptosis and smaller lesion area in *LDLR*^*−/−*^ mice after 5 or 12 weeks on a Western diet.^47^ Given the variability in genetic mouse models, diets used, and stage of atherosclerosis examined, a direct comparison to the current study is not possible. However, in the current study, in which lesions were assessed in dKO mice on a chow diet at later times (25 and 40 weeks) than the above studies (5 to 20 weeks on high‐fat or Western diets), data are consistent with the hypothesis that elevated macrophage apoptosis leads to reduced atherosclerosis at early stages and increased atherosclerosis at later stages of lesion growth. Furthermore, given that the model used in our study was global ablation of the *TDAG51* gene, the observed reduction in lesion size may not be solely attributable to decreased macrophage apoptosis; other cell types and mechanisms may be involved.

Previous studies have indicated that PPARγ acts as a positive regulator of antioxidant defenses.^48^ Consistent with the inverse correlation between *TDAG51* deficiency and PPARγ expression, rosiglitazone treatment induced Prdx‐1 protein expression by 23% (*P*<0.05) in *TDAG51*^*−/−*^ macrophages, compared with 12% (*P*<0.05) in wild‐type macrophages (Figure 14F). These findings are consistent with previous studies showing that 15d‐PGJ_2_, a natural PPARγ agonist, induces expression of antioxidant proteins including Prdx‐1^49^ and supports the hypothesis that the increased Prdx‐1 expression associated with *TDAG51* deficiency is a consequence of elevated expression and/or activity of PPARγ, leading to the reduced atherosclerotic lesion growth observed in the dKO mice. Given the ability of TDAG51 to modulate transcriptional activity,^13^ we are currently investigating the possibility that TDAG51 modulates PPARγ transcriptional activity and/or nuclear localization.

It is well established that PPARγ activates reverse cholesterol transport in lesion‐resident macrophages.^37–38^ Furthermore, PPARγ ligands promote the reduction of atherosclerotic lesions,^50–51^ whereas the conditional knockout of macrophage PPARγ enhances atherosclerosis without altering plasma lipid levels.^52^ We observed increased cholesterol efflux as well as increased expression of ABCG1 in *TDAG51*^*−/−*^ peritoneal macrophages. *TDAG51*^*−/−*^ macrophages, compared with wild‐type cells, also accumulated fewer lipids, suggesting a reduction in foam cell formation. We recently reported that *TDAG51* deficiency induced age‐associated adipogenesis and hepatic steatosis in *TDAG51*^*−/−*^ mice,^18^ further implicating PPARγ in modulating the effects of *TDAG51* deficiency. Taken together, our results suggest that *TDAG51* deficiency, despite its effects on adipogenesis and hepatic lipogenesis, reduces atherosclerotic lesion growth via activation of multiple cellular pathways that regulate apoptosis, antioxidant status, and lipid storage/export.

Although studies reporting the beneficial role of PPARγ agonists in mitigating atherogenesis have been fairly consistent in mouse models, some controversy exists over the cardiovascular risk associated with the use of thiazolidinediones (TZDs) in patients. Several reports have indicated rosiglitazone is associated with significantly increased risk of myocardial infarction.^53–54^ In contrast, others have found the evidence for an association between rosiglitazone and myocardial infarction and CVD mortality to be inconclusive.^55^ Furthermore, pioglitazone, another PPARγ agonist, was reported to have significantly reduced cardiovascular risk in the PROactive study in diabetic patients with preexisting CVD.^56^ Given the current debate over the potential beneficial and adverse events associated with TZDs in regard to CVD risk, it remains to be determined whether increased PPARγ associated with *TDAG51* deficiency and decreased atherosclerotic lesion growth in a mouse model can be extrapolated to a clinical setting. It should also be noted that this mechanism is likely not the only one that drives the antiatherogenic effect of *TDAG51* deficiency. This could reflect TDAG51's role in apoptosis or signaling pathways that modulate macrophage viability and lipid metabolism.

The genetic association results from 2 independent case–control studies^31,30^ suggest that genetic variants in the *TDAG51* region are associated with CVD. This finding is intriguing given the biological evidence for this gene in atherosclerosis. However, meta‐analyses of genomewide association studies^57–58^ did not find an association between SNPs in the *TDAG51* region and CVD at the genomewide significance level (*P*<5×10^−8^). Therefore, the genetic association results we report here may have overestimated the risk associated with the SNPs we examined. Additional studies are thus required to further evaluate whether genetic variations in the *TDAG51* gene are associated with altered expression of this gene and CVD. Functional characterization of these SNPs will also be helpful to understand the potential role of these SNPs in CVD. Because rs2367446 is in an intergenic region 20 kbp from *TDAG51*, it is possible that this SNP affects transcription of the *TDAG51* gene.

In summary, we have characterized a previously unknown role for *TDAG51* in modulating atherosclerotic lesion development and progression while addressing the underlying cellular mechanisms by which *TDAG51* deficiency modulates these processes. Because the effects of *TDAG51* are mediated by multiple pathways that affect lesion development and stability, our findings provide a unique opportunity to develop novel therapeutic approaches that decrease the risk of CVD by targeting TDAG51 expression and/or activity.

## Source of Funding

This work was supported, in part, by research grants to Richard C. Austin from the Heart and Stroke Foundation of Ontario (PRG‐6502), the Canadian Institutes of Health Research (MOP‐126083, MOP‐111239), and the Ontario Research and Development Challenge Fund. Financial support from St. Joseph's Healthcare Hamilton is acknowledged. Jeffrey G. Dickhout is supported by the St. Joseph's Healthcare Hamilton Division of Nephrology Junior Research Award. Richard C. Austin is a Career Investigator of the Heart and Stroke Foundation of Ontario and holds the Amgen Canada Research Chair in the Division of Nephrology at St. Joseph's Healthcare and McMaster University.
